# Conotoxins from sea snails as potential bone remodeling disruptors

**DOI:** 10.1093/jbmrpl/ziaf025

**Published:** 2025-02-10

**Authors:** Brenda Iduarte-Frias, Pierrick G J Fournier, Pavel Galindo-Torres, Claudia Ventura-López, Alexei F Licea-Navarro, Johana Bernáldez-Sarabia, Patricia Juárez

**Affiliations:** Life Sciences Graduate Program, Biomedical Innovation Department, Centro de Investigación Científica y de Educación Superior de Ensenada (CICESE), Ensenada, Baja California 22860, México; Biomedical Innovation Department, Centro de Investigación Científica y de Educación Superior de Ensenada (CICESE), Ensenada, Baja California 22860, México; Biomedical Innovation Department, Centro de Investigación Científica y de Educación Superior de Ensenada (CICESE), Ensenada, Baja California 22860, México; Aquaculture Department, Centro de Investigaciones Biológicas del Noroeste (CIBNOR), La Paz, Baja California Sur 23096, México; Marine Biotecnology Department, Centro de Investigación Científica y de Educación Superior de Ensenada (CICESE), Ensenada, Baja California 22860, México; Biomedical Innovation Department, Centro de Investigación Científica y de Educación Superior de Ensenada (CICESE), Ensenada, Baja California 22860, México; Biomedical Innovation Department, Centro de Investigación Científica y de Educación Superior de Ensenada (CICESE), Ensenada, Baja California 22860, México; Biomedical Innovation Department, Centro de Investigación Científica y de Educación Superior de Ensenada (CICESE), Ensenada, Baja California 22860, México

**Keywords:** sea snail, *conus*, α-conotoxin, bone remodeling, osteoclast, osteoblast

## Abstract

The ocean provides food and shelter to diverse marine species, and it is an exceptional source of potential bioactive natural products with promising medicinal properties. Among these, α-conotoxins from venom sea snails show tremendous potential. Our study characterized the effects of synthetic α-conotoxins, sXm1b and sVc1.1, on bone remodeling. Transcriptomic analysis showed significant modulation of critical biological processes, leading to increased osteoclast activity and decreased osteoblast mineralization. sXm1b and sVc1.1 treatment also promoted genes involved in osteoblast and osteoclast proliferation. Interestingly, sVC1.1 showed higher osteoclast gene modulation and reduced the expression of genes critical for osteoblast development and differentiation. In vitro, functional evaluations demonstrated increased osteoclastogenesis and resorption, along with decreased differentiation and mineralization by osteoblasts. In a 3D ex vivo calvaria culture model, these conotoxins significantly decreased bone area, increased osteoclast number, and modulated the expression of osteoclast- and osteoblast-related genes. The findings highlight the promise of α-conotoxins as modulators of bone remodeling for treating non-genetic bone mass accumulation problems while also cautioning about potential adverse effects on bone in individuals undergoing conotoxin therapy for pain management.

## Graphical Abstract

**Graphical Abstract:**
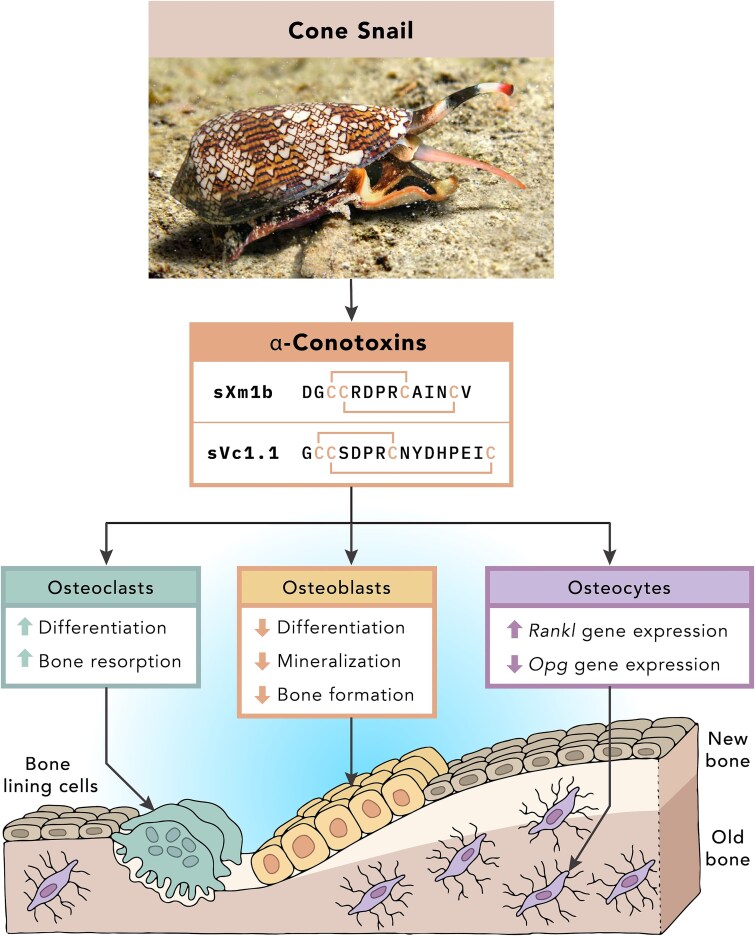

## Introduction

Bone remodeling is a tightly coupled process, which maintains bone strength and mineral homeostasis. Osteocytes, osteoblasts, and osteoclasts must communicate with one another in a complex way during this process. The strict regulation of osteoclast resorption and osteoblast bone formation is necessary to maintain bone integrity. Imbalances in this equilibrium can lead to pathological conditions characterized by altered bone metabolism. Bone loss occurs when bone formation is less than resorption, while anabolic processes occur when bone formation exceeds resorption.^1^ Despite the scientific advances, there is still a need for new therapies against pathologies that affect bones (osteoporosis, bone metastasis, osteoarthritis, and osteosarcoma).

Sea snails of the genus *Conus* are slow-moving organisms that have adapted to an environment where their prey moves quickly. They have developed a venom apparatus to capture their prey or defend themselves against predators to survive. The venom of cone snails is highly efficient because of its principal components: small toxic peptides known as conotoxins.^2^ Conotoxins are a diverse group of small peptides with 10-45 amino acids that interact with ligand-gated and voltage-gated ion channels, G protein-linked receptors, nicotinic acetylcholine receptors, and norepinephrine transporters.^2–4^ Conotoxins possess high selectivity and affinity for their targets, making them attractive pharmacological tools for developing novel drugs. Conotoxins have been studied as potential treatments for Parkinson’s disease, Alzheimer’s disease, epilepsy, cancer, type 2 diabetes, and chronic pain.^5–8^ MVIIA, a synthetic VI-conotoxin, has been approved for the treatment of intense and persistent pain.^9^^,^^10^ The α-conotoxins are a group of pharmacological compounds that specifically interact with nicotinic acetylcholine receptors (nAChRs). These peptides usually have 12-20 amino acid residues and specifically differentiate between muscle and neuronal nAChR subtypes, considering their composition as homo- or hetero-pentamers of α, β, γ, δ, or ε subunits.^11^^,^^12^ The α-conotoxin Vc1.1 has been tested in phase I and IIA clinical trials to assess its efficacy in treating neuropathic pain. Remarkably, the drug administered subcutaneously had no adverse effects. Researchers found that Vc1.1 was less effective in humans than in rats due to variations in the α9α10 nAChR isoform. This disparity has stopped the development of Vc1.1 for pain therapy.^13^^,^^14^

nAChRs were originally assumed to be expressed only in neuron and muscle cells. New research shows that nAChRs are broadly expressed in mammalian cells, including in osteoblasts,^15–17^ osteocytes,^18^ and osteoclasts.^19^^,^^20^ Due to their specificity, α-conotoxins are effective in regulating nAChR function and the associated biological processes. α-conotoxins are interesting tools for studying bone biology as they are target-specific biochemical pathways involved in bone remodeling.

**Figure 1:**
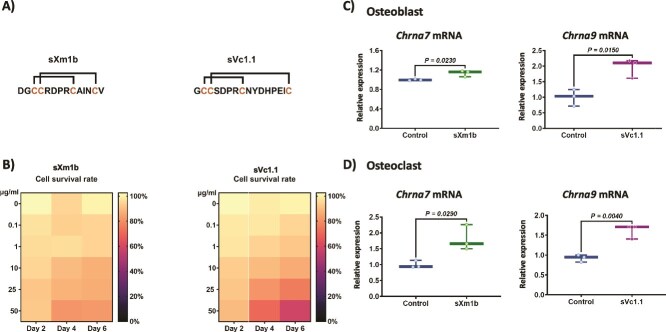
Impact of conotoxin treatment on cell viability and nicotinic acetylcholine receptors expression. (A) Sequences of the synthetic conotoxins sXm1b and sVc1.1. Connecting lines indicate disulfide bonds. (B) Cell viability of bone marrow cells cultivated with or without sXm1b and sVc1.1 conotoxins measured by MTT assay. The heatmap shows cell viability at 0.1-50 μg/mL conotoxin doses after 2, 4, and 6 d of treatment. Cell viability is represented on a relative intensity scale, higher values correspond to increased absorbance. Results are shown as mean ± SEM absorbance at 570 nm. One-way ANOVA and Dunnett’s multiple comparisons were used for statistical analysis. Expression of nicotinic acetylcholine receptor in (C) osteoblasts and (D) osteoclasts. Total RNA was collected from each sample, and gene expression was measured by qRT-PCR and standardized to *Rpl32.* The relative expression levels were calculated by first normalizing the Ct values of the target genes to the reference gene RPl32 (ΔCt), followed by normalization to the control condition (ΔΔCt). The statistical analysis was performed using Mann–Whitney U test.

Vc1.1 was originally isolated from the venom of *Conus victoriae*, and Xm1b from *Conasprella ximenes* are specific antagonists of the α9α10 nAChR,^21^ and the α7 nAChR,^22^ respectively. Although bone cells express these receptors, the effects of these conotoxins in bone cells are unknown. In this study, we aimed to determine whether the synthetic conotoxins sVc1.1 and sXm1b (Figure 1A) can modulate bone remodeling using in vitro and ex vivo models involving osteoclasts, osteoblasts, and osteocytes.

## Materials and methods

### Animals

Animal protocols were performed by the Federal Regulation for Animal Experimentation and Care (SAGARPA; NOM-062-ZOO, 1999, Mexico) at the Center of Scientific Research and Higher Education of Ensenada (CICESE). BALB/c mice (Balb/CAnNHsd) were obtained from Envigo and bred at the CICESE. Mice received water and food (2018 Teklad Global 18% protein rodent diet; Teklad) ad libitum and were housed under a 12-h light/night cycle. They were acclimated for at least 2 wk before starting the experiments.

### Conotoxins

α-Conotoxin sVc1.1 was discovered by Sandall et al.^23^ from the venom ducts of *C. victoriae* using a PCR screen of cDNAs. Conotoxin sXm1b was isolated and characterized from *C. ximenes* in 2013 by Bernáldez-Sarabia.^22^ This study used a synthetic form of each conotoxin purchased from Ontores Biotechnologies Co. Ltd. The toxins were solubilized in sterile distilled water.

### Cell viability assay

Cell viability was determined using the 3-(4,5-dimethylthiazol-2-yl)2,5-diphenyltetrazolium bromide (MTT) dye reduction assay. Bone marrow cells isolated from the hindlimbs (tibia and femur) of 6- to 8-wk-old BALB/c mice were resuspended in red blood lysis buffer (155 mM NH_4_Cl, 10 mM NaHCO_3_, 1 mM EDTA) to remove erythrocytes. Then, bone marrow cells were cultured in α-minimum essential medium (α-MEM) supplemented with 10% fetal bovine serum (FBS, Biowest), 1% antibiotic/antimycotic (Ab/Am, Corning), 25 ng/mL murine macrophage colony-stimulating factor (M-CSF, PeproTech), and conotoxins (0.1-50 μg/mL) and incubated at 37 °C in a humidified 5% CO_2_ atmosphere. Cell viability was evaluated on days 2, 4, and 6 of culture, and MTT (Sigma) was added at a concentration of 5 mg/mL. After 4 h of incubation at 37 °C, the cell reaction was stopped by adding an HCl (10 mM)-SDS (10% w/v) solution and incubated overnight at 37 °C. The absorbance at 570 nm was measured using a spectrophotometer (Epoch, BioTek).

### Osteoclast differentiation assays

Femurs and tibias were collected immediately after euthanasia to isolate bone marrow cells from female Balb/c mice (6-8 wk). The ends of the bones were pierced with a 1 mL syringe, and the bone marrow was flushed with PBS. The cell suspension was filtered through a 70 μm nylon cell strainer (Corning) and centrifuged. The obtained cells were suspended in red blood cell lysis buffer (BioLegend). The cell pellet was resuspended in α-MEM supplemented with 10% FBS, 1% Ab/Am, and 50 ng/mL of M-CSF and cultured for 48 h under standard conditions. In 96-well plates, 4 × 10^4^ adherent cells (osteoclast precursors) were cultured in α-MEM containing 10% FBS, 1% Ab/Am, 25 ng/mL of M-CSF, 25 ng/mL of recombinant murine RANKL (PeproTech), and 100 ng/mL of synthetic conotoxin. Osteoclasts differentiated without conotoxins were used as the positive controls. The cells were cultured for seven days at 37 °C in a humidified atmosphere with 5% CO_2_, and half of the medium was renewed every 2 d. Osteoclasts were stained for tartrate-resistant acid phosphatase (TRAP) activity (Sigma-Aldrich), and the number of osteoclasts (TRAP^+^, multinucleated cells) was counted using a Zeiss Axio Scope A1 microscope (Zeiss). Resorption activity was evaluated on Osteo Assay Surface Stripwells (Corning), and images were collected using a Zeiss Axio Scope A1 microscope with an Axiocam 505 color (Zeiss). The cells were harvested and processed for RNA extraction.

Human osteoclasts were obtained from the differentiation of human peripheral blood mononuclear cells (PBMCs). Venous whole blood (22 mL) was drawn from each healthy male or female volunteer by venipuncture and stored in EDTA tubes (BD) for further processing. Blood was diluted 1:1 with PBS, layered over Ficoll-Paque (Cytiva), and centrifuged for 30 min at 800 × *g*. The cell layer of PBMCs was collected in a clean 50 mL conical tube, washed in PBS, and resuspended in red blood cell lysis buffer (BioLegend). The supernatant was discarded, and the cell pellet was resuspended in α-MEM (10% FBS, 1% Ab/Am). PBMCs were seeded at a density of 750 000 cells/cm^2^ in α-MEM (10% FBS, 1% Ab/Am) supplemented with 25 ng/mL human macrophage colony-stimulating factor (PeproTech) for 72 h to generate M-CSF-dependent macrophages. Cells (4 × 10^4^/well) were added to a 96-well plate and incubated in a total volume of 200 μL of α-MEM (10% FBS, 1% Ab/Am) medium containing 25 ng/mL M-CSF, 25 ng/mL recombinant human RANKL (PeproTech), and 100 ng/mL of synthetic conotoxin for 9 d at 37 °C in 5% CO_2_ in humidified air. Every 2- or 3-d, half of the media was renewed every 2 or 3 d. Osteoclasts were stained for TRAP activity (Sigma-Aldrich), and the number of osteoclasts (TRAP^+^, multinucleated cells) was counted using a Zeiss Axio Scope A1 microscope (Zeiss).

### Osteoblast differentiation and mineralization assay

Bone marrow cells were collected by flushing the long bones of female Balb/c mice (6-8 wk) and maintained for 3 d in α-MEM containing 10% FBS and 1% Ab/Am at 37 °C in a humidified incubator containing 5% CO_2_. To promote osteoblastic differentiation and mineralization, cells (8 × 10^4^ cells/well) were plated in 96-well plates in osteogenic α-MEM containing 10% FBS, 1% Ab/Am, 25 μg/mL ascorbic acid (Sigma), and 10 mM β-glycerophosphate (Sigma). Synthetic conotoxins (100 ng/mL) were added to the osteogenic medium, and half of the medium was refreshed every 3 d for 21 d. Alizarin red staining (Sigma) was performed on osteoblasts to stain calcified areas. The cells were fixed with 70% ice-cold ethanol (Sigma) for 1 h and rinsed twice with deionized water. Mineralized osteoblasts were incubated in alizarin red staining solution (40 mM) for 35 min at room temperature. After several washes with deionized water, the cells were air-dried, and representative images were collected using a Zeiss Axio Scope A1 microscope with an Axiocam 505 color camera. The cells were then incubated on a shaker in a 10% (v/v) acetic acid (Sigma) solution for 30 min at room temperature. Supernatants were collected, mineral oil (Sigma-Aldrich) was added to avoid evaporation, and then heated at 85 °C for 10 min in a water bath. Samples were transferred to ice for 10 min and centrifuged for 15 min at 20 000 × *g*. Supernatant was neutralized by adding 10% NH_4_OH solution (Sigma-Aldrich), transferred to a 96-well plate in triplicates, and absorbance at 405 nm was measured. When differentiated for RNA isolation, cells were cultured in 24-well plates at a density of 9.5 × 10^5^ cells/well.

### Osteocyte-enriched bone preparations

Hindlimbs were collected immediately after euthanasia; from female BALB/c mice 8-wk-old, the muscle, ligaments, tendons, and periosteum were meticulously removed. Epiphyses were cut, and the bone marrow was flushed with PBS until visibly clean. Bones were subjected to serial digestion with collagenase and EDTA, as previously described.^24^ The bone pieces were treated with synthetic conotoxins (100 ng/mL) in α-MEM containing 10% FBS and 1% Ab/Am at 37 °C in a humidified incubator containing 5% CO_2_. After 72 h, osteocyte-enriched bones were homogenized (FastPrep24, MP Biomedicals) in TRI Reagent (Sigma) and processed for RNA extraction according to the manufacturer’s instructions.

### Hemicalvaria ex vivo culture

BALB/c mice were sacrificed by decapitation at postnatal day 4, and calvarial tissue was collected, as previously reported.^25^ For synthetic toxin treatment, hemicalvarias were incubated in α-MEM containing 10% FBS, 1% Ab/Am, and an addition of 100 ng/mL of conotoxin sXm1b or sVc1.1. The medium was changed every 2 d after 7 d in culture, and hemicalvarias were processed for RNA extraction or histology.

### Bone histology and histomorphometry

The hemicalvarias were immersed in a solution of 10% buffered formalin and kept at a temperature of 4 °C for a duration of 24 h. They were then subjected to decalcification in a solution of 10% (w/v) EDTA at 4 °C for 48 h. Finally, the hemicalvarias were embedded in paraffin wax to facilitate sectioning. Sections with a thickness of 4 μm were obtained from the hemicalvarias using a Microm HM 355S microtome (Thermo Fisher Scientific). The tissue sections underwent staining with H&E and Orange G and were then processed for histomorphometric examination. The section images were acquired using a Zeiss Axio Scope A1 microscope equipped with an Axiocam 505 color camera. The histomorphometric analysis was conducted in a blinded manner, where tissue slices were assigned a random number prior to examination using ImageJ software.

### Gene expression analysis by qRT-PCR

RNA was isolated from osteoblasts, osteoclasts, and hemicalvaria using the RNeasy Mini Kit (Qiagen) following the manufacturer’s instructions. The process of converting 500 ng of total RNA into complementary DNA (cDNA) was achieved by utilizing anchored oligo dT primers and SuperScript II reverse transcriptase (Invitrogen). The cDNAs were examined in triplicate using quantitative real-time PCR using a 7500 Real-Time PCR System (Thermo Fisher Scientific). qRT-PCR was conducted using QuantiTect SYBR Green Master Mix (Qiagen). Gene expression was measured by quantifying the amount of gene-specific cDNA using standard curves. The relative amounts were then adjusted based on the expression of the reference gene *Rpl32*. Data analysis was done using the delta-delta Ct (ΔΔCt) method. The sequences of the oligonucleotides used are listed in Table 1.

**Table 1:** Sequences of oligonucleotides used for qRT-PCR analysis.

**Gene**	**Gene ID**	**Forward primer (5′ to 3′)**	**Reverse primer (5′ to 3′)**
*Rpl32*	19951	CAGGGTGCGGAGAAGGTTCAAGGG	CTTAGAGGACACATTGTGAGCAATC
*Chrna7*	11441	CACATTCCACACCAACGTCTT	AAAAGGGAACCAGCGTACATC
*Chrna9*	231252	GGAACCAGGTGGACATATTCAAT	GCAGCCGTAGGAGATGACG
*Nfatc1*	18018	GGAGAGTCCGAGAATCGAGAT	TTGCAGCTAGGAAGTACGTCT
*Oscar*	232790	CCTGGCACCTACTGTTGCTA	TGGAGTTGATGTCCCCTGGT
*Trap*	11433	CTGGTATGTGCTGGCTGGAA	CCCAGGTCTCGAGGCATTTT
*Ctsk*	13038	GAGGGCCAACTCAAGAAGAA	GCCGTGGCGTTATACATACA
*Rank*	21934	TGTGTGTTGCTCCCACATTT	TCCCATGGAGCTGGAGTTAC
*Runx2*	12393	GGCACAGACAGAAGCTTGATGA	GAATGCGCCCTAAATCACTGA
*Alpl*	11647	CACAGATTCCCAAAGCACCT	GGGATGGAGGAGAGAAGGTC
*Col1a1*	12842	CCTGGATGCCATCAAAGTCT	CGCCATACTCGAACTGGAAT
*Bglap*	12096	CTTGGCTTCTGACTGGGTGT	GGCCACTTACCCAAGGTAGC
*Rankl*	21943	TGTGTGTTGCTCCCACATTT	TCCCATGGAGCTGGAGTTAC
*Opg*	18383	CCAAGCATGCTGAGTGAGAA	CAGCTGTGAGGAGAGGAAGG
*Dmp1*	13406	CATTCTCCTTGTGTTCCTTTGGG	TGTGGTCACTATTTGCCTGTG
*Mepe*	94111	GTCTGTTGGACTGCTCCTCTT	CACCGTGGGATCAGGATACA
*Sost*	74499	AGCCTTCAGGAATGATGCCAC	CTTTGGCGTCATAGGGATGGT

### RNA extraction, sequencing, and data analyses

Osteoclasts and osteoblasts were differentiated in 6-well plates as described. Total RNA from 3 independent cultures was isolated at days 7 and 21 using the RNeasy Mini Kit (Qiagen) according to the manufacturer’s instructions. The total RNA (1 μg per sample) was quantified using a Qubit 3 fluorometer (Invitrogen). RNA integrity was analyzed using a bioanalyzer (Agilent). Only RNA with an RNA integrity number (RIN) above 8 was used for library construction using TruSeq Stranded mRNA kit (Illumina) following the manufacturer’s instructions. The average size and concentration were validated using Bioanalyzer and Qubit, respectively. The 30 libraries were sequenced using the HiSeq (Illumina) platform sequencer at the Novogene corporation to obtain 20 million reads per library with a 2 × 150 nt read length.

Raw reads were initially assessed using the FastQC software (http://www.bioinformatics.babraham.ac.uk/projects/fastqc/). Subsequently, Illumina adapters, indices, and low-quality reads were removed using Trimmomatic 0.39v.^26^^,^^27^ Reads with a phred33 score <30 and shorter than 36 bp in length were removed. To summarize the FastQC results before and after adapter processing, we employed MultiQc software.^28^ Trimmed paired reads from each library were pseudoaligned to the *Mus musculus* transcriptome (GRmc39) using Kallisto V.0.46.1.^29^ We used the web-based differential gene expression analysis tool and integrated Differential Expression and Pathway analysis (iDEP.92).^30^ We retained genes with minimal counts per million (CPM) ≥1 in at least one library. Differential expression analysis was performed using the DEseq2 package.^31^ Genes with an FDR cutoff <0.1 and Log2FC > 2 were kept. The remaining analyses were performed using default parameters in iDEP.92 (https://idepsite.wordpress.com/). Finally, heatmap plots were done using the R package ComplexHeatmap.^32^

### Statistical analysis

Statistical analyses were performed using GraphPad Prism software (v9.0; GraphPad Software, Inc.). Statistical analyses of the 2 groups were conducted using a two-tailed Student’s *t*-test or Mann–Whitney test. Comparisons of three or more groups were conducted using a one-way ANOVA, followed by Dunnett’s post-test. Results are presented as box plots with median and interquartile range; all data points and *p* ≤ .05 were considered significantly different.

## Results

### In vitro *safety-profile of conotoxins on bone marrow cells*

First, we performed an MTT experiment to determine if synthetic conotoxin therapy is cytotoxic to bone cells. Mouse bone marrow cells were cultured in increasing concentrations of the conotoxins sXm1b and sVc1.1 (Figure 1A) for up to 6 d. Conotoxin treatment for 2 d did not significantly affect cell viability compared to the control group (Figure 1B). However, both conotoxins reduced cell viability starting at concentrations of 25 μg/mL after 4 d of treatment. Treating with the conotoxins for 6 d led to a further concentration-dependent decrease in cell viability, with a maximum reduction of 30% for sVc1.1 at 50 μg/mL, and 40% for sXm1b at 50 μg/mL. However, concentrations of conotoxins lower than 1 μg/mL did not significantly impact cell viability compared to the control conditions. Thus, we use a non-toxic concentration of 100 ng/mL of conotoxins in the following experiments.

Next, we confirmed the expression of the α7 and α9 subunits of the nAChR, *Chrna7,* and *Chrna9*, respectively. These receptors serve as molecular targets for the conotoxins sXm1b and sVc1.1, respectively. Mouse bone marrow cells were cultivated in osteoclastogenic and osteoblastogenic conditions and treated with 100 ng/mL of conotoxins. Once each cell differentiation process was complete, we evaluated the expression of these nicotinic receptors. Results confirmed the expression of α7 and α9 nAChR in osteoblast and osteoclast cell cultures and that their expression was significantly increased in the presence of the conotoxins (Figure 1C and D).

### Conotoxin treatment induces transcriptomic changes in osteoclasts and osteoblasts

We used transcriptome analysis as an initial exploratory step to identify potential pathways and processes impacted by conotoxins in mouse osteoclasts and *osteoblasts* in vitro*.* We obtained transcriptome data sets for in vitro-differentiated bone cells treated with conotoxins. Osteoclast samples were collected on day 7, while osteoblast samples were collected on day 21. All replicates passed quality control and were analyzed to identify genes with differential expression during osteoclast and osteoblast differentiation treated with conotoxins.

**Figure 2:**
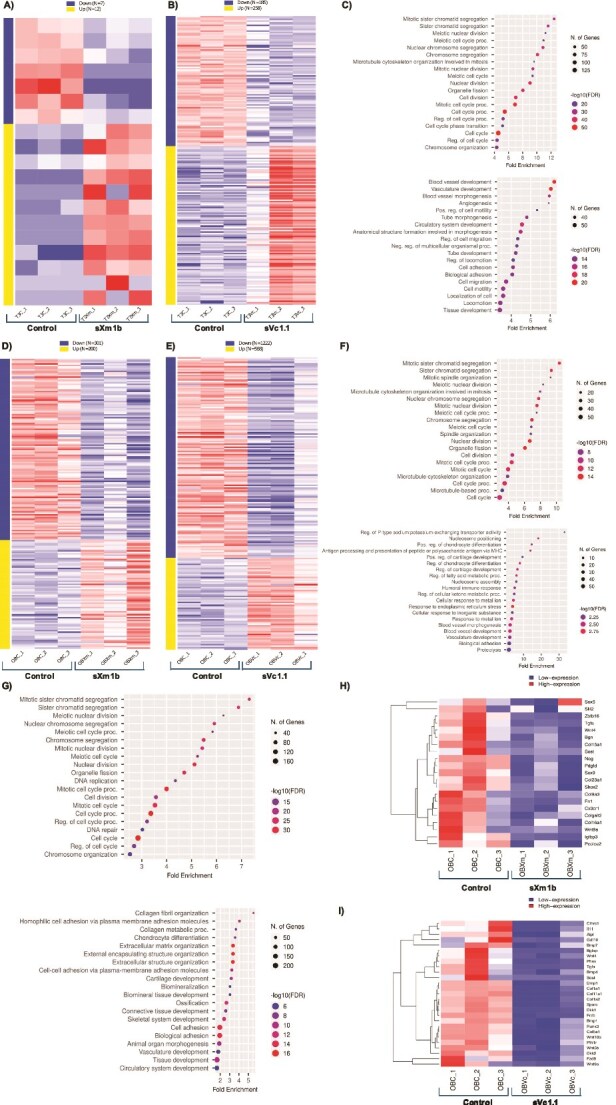
Conotoxins induce transcriptomic changes in osteoclasts and osteoblasts. RNAseq analysis of differentially expressed genes of conotoxin-treated osteoclasts (7 d) (A and B) and osteoblasts (21 d) (D and E). Dot plot visualization of biological process up- and down-regulated GO keywords in osteoclasts treated with sVc1.1 (C) and osteoblasts (F and G). The size of the dots shows the number of genes enriched in each biological process, while the color denotes the false discovery rate (FDR < 0.1) (H) and (I) osteoblast-related genes were expressed (Log2FC > 2) in the heat map. Defined genes with FDR < 0.1 and Log2FC > 2 using DEseq2, and all other analyses utilized default values in iDEP.92.

Transcriptomic analysis of osteoclasts (Figure 2A) showed the differential expression of 19 genes (7 downregulated and 12 upregulated) in the cells treated with conotoxin sXm1b compared to control. Osteoclasts treated with conotoxin sVc1.1 showed significant differential expression of 443 genes (185 downregulated and 258 upregulated) when compared to the control group (Figure 2B). To understand the functional significance of the differentially expressed genes, we conducted a Gene Ontology (GO) enrichment analysis. The analysis revealed no associations with biological processes among the differentially expressed genes in osteoclasts treated with the conotoxin sXm1b. However, osteoclasts treated with the conotoxin sVc1.1 (Figure 2C) showed upregulated genes were predominantly associated with biological processes such as “cell cycle” (*n* = 127 genes), “cell division” (*n* = 74 genes), and “regulation of the cell cycle” (*n* = 69 genes). In contrast, downregulated genes were primarily related to “cellular response to chemical stimulus” (*n* = 74 genes), “circulatory system development” (*n* = 51 genes), “vasculature development” (*n* = 46 genes), and “blood vessel development” (*n* = 45 genes). These findings suggest that the conotoxin sVc1.1 can modulate gene expression in osteoclast cultures and biological processes related to bone biology.

Furthermore, transcriptomic analysis of osteoblasts treated with sXm1b conotoxin indicated the differential regulation of 501 genes (301 downregulated and 200 upregulated) (Figure 2D). Similarly, the conotoxin sVc1.1 (Figure 2E) significantly modulated the expression of 1790 genes (1222 downregulated and 568 upregulated). The most prevalent signaling pathways affected by both toxins involve genes related to skeletal system development, chondrocyte differentiation, connective tissue, and collagen organization. GO analysis showed that osteoblasts treated with the conotoxin, sXm1b (Figure 2F) primarily exhibited upregulated changes in “cell cycle” (*n* = 58 genes), “cell cycle process” (*n* = 49 genes), and “cellular stress response” (*n* = 42 genes). Conversely, downregulated processes were associated with “blood vessel development” (*n* = 32), “vasculature development” (*n* = 32 genes), “cartilage development” (*n* = 12 genes), and “chondrocyte differentiation” (*n* = 10 genes). Similarly, the conotoxin sVc1.1 (Figure 2G) led to the upregulation of the genes associated with “cell cycle” (*n* = 162 genes), “cell cycle process” (*n* = 133 genes), and “cellular stress response” (*n* = 120 genes). Besides, there was also downregulation of enriched terms related to “cell adhesion” (*n* = 170 genes), “skeletal system development” (*n* = 92), “ion membrane transport” (*n* = 85 genes), “ossification” (*n* = 77 genes), “cartilage development” (*n* = 47), “osteoblast differentiation” (*n* = 40 genes), “bone development” (*n* = 38 genes), “chondrocyte differentiation” (*n* = 30 genes), and “collagen metabolic process” (*n* = 28 genes). GO analysis indicated a substantial difference in osteoblast transcriptomes treated with the 2 conotoxins, specifically in downregulated gene expression. We analyzed the effects of the conotoxins on cell proliferation and the cyclin gene expression of osteoblast and osteoclast precursors, Table S1 (supplementary data) details the specific gene expression changes observed. Our analysis revealed significant changes in the expression levels of genes related to cell proliferation, as well as the cell cycle in osteoblast and osteoclast cultures treated with both conotoxins. sVc1.1 treatment upregulated key cell cycle regulatory genes, including *Foxm1*, regulators of mitosis (Cdc20 and Cdc25b), and transcription factors that control entry into the S phase of the cell cycle (E2f and E2f2), suggesting that this conotoxin stimulates cell growth. In addition, we observed increased expression of genes associated with proliferation, including *Mcm5*, *Aurkb*, *Prc1*, *ki67*, and *Birc5*. Increased expression of the KIF family genes further suggests enhanced mitotic and proliferative activity. While conotoxins sXm1b and sVc1.1 promoted osteoblast proliferation, our data also indicate a decrease in the expression of genes critical for osteoblast development and differentiation. Notably, genes associated with the Wnt pathway (*Wnt4* and *Wnt9a*) and transcription factors essential for osteoblast differentiation (*Sox5*, *Sox6*, and Sox9) were downregulated in treated cells.

*In*  *silico* analysis showed that treatment with the conotoxin sXm1b (Figure 2H) modifies the expression of genes associated with the extracellular matrix of bone and cartilage development (*Pcolce2*, *Col9a3*, *Col15a1*, *Col16a1*, *Bgn*, and *Fn1*), bone development and osteoblast activity (*Nog*, *Wnt9a*, *Wnt4*, *Sost*, and *Tgfa*), and chondrogenesis and early skeletal development genes (*Sox5*, *Sox9*, and *Shox2*). Additionally, conotoxin sVc1.1 (Figure 2I) negatively regulated the expression of genes involved in osteoblast differentiation and activity (*Alpl*, *Bglap*, *Dmp1*, *Phex*, *Col1a1*, and *Col1a2*), osteoblast regulation (*Pth1r*, *Sost*, *Dkk1*, *Dkk2*, *Wnt4*, *Wnt5b*, *Wnt9a*, *Wnt10b*, and *Fzd9*), and skeletal development (*Il11*, *Tgfa*, *Bmp7*, *Bmp4*, and *Bmp1*). These data indicate that conotoxins can regulate osteoblast differentiation and bone remodeling genes.

### Conotoxin treatment decreases osteoblast function while increasing osteoclast function *in vitro*

**Figure 3:**
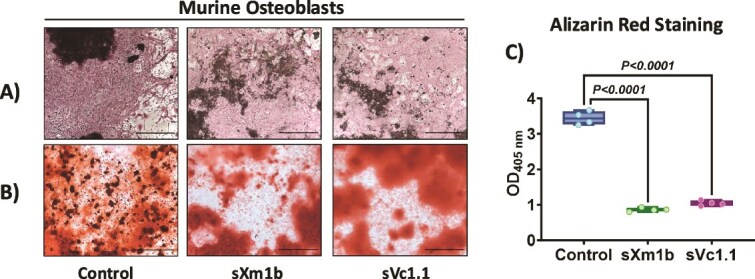
Synthetic conotoxins decrease osteoblast activity *in vitro*. Bone marrow cells were cultivated in osteogenic conditions (25 μg/mL ascorbic acid, 5 mM β-glycerophosphate) in the presence or absence of conotoxins (100 ng/mL). After 21 d of culture, alkaline phosphatase (A) and alizarin red (B) staining were performed. Alizarin red staining was quantified at 405 nm (C).

Next, we used mouse bone marrow cells and human osteoclast precursors to determine the effects of these conotoxins on osteoblastogenesis and osteoclastogenesis in vitro. Conotoxins sXm1b and sVc1.1 were evaluated on mouse bone marrow cells cultured in the presence of ascorbic acid and β-glycerophosphate to determine their impact on osteoblastogenesis. As shown by alkaline phosphatase staining, osteoblast activity was not affected by conotoxin treatment (Figure 3A). While alizarin red staining indicated the presence of mineralized nodules in the presence and absence of conotoxins (Figure 3B), a quantitative analysis of the staining intensity indicated that both conotoxins significantly inhibited osteoblast mineralization (Figure 3C).

**Figure 4:**
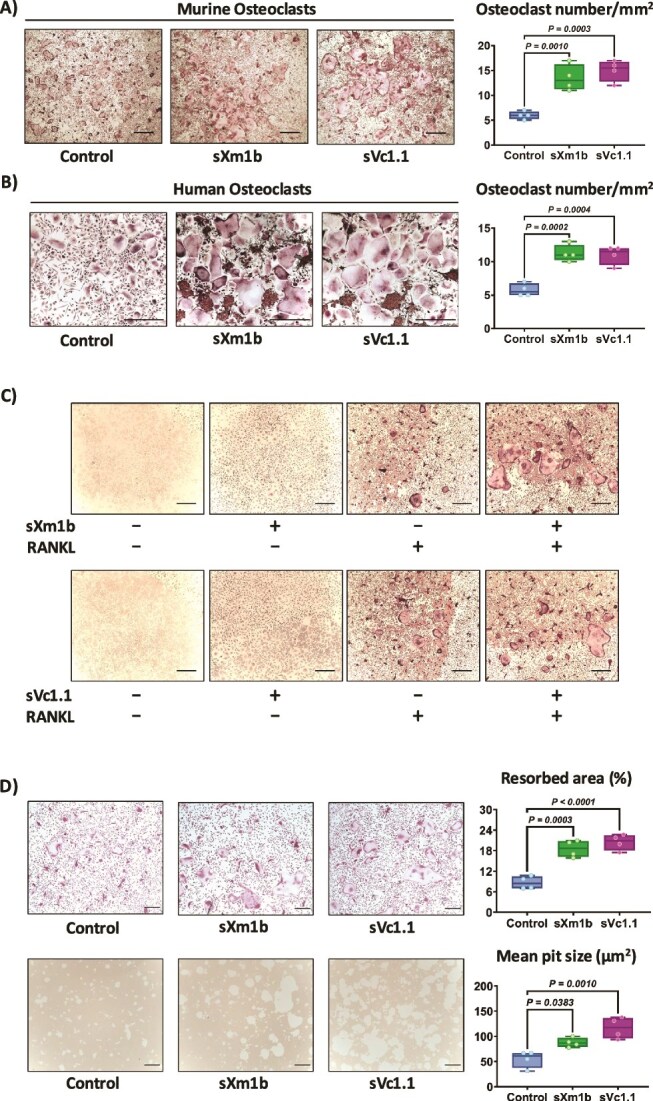
Synthetic conotoxins enhance RANKL-mediated osteoclast differentiation and resorption in vitro. (A) Mouse bone marrow cells were cultivated with M-CSF (25 ng/mL) and RANKL (25 ng/mL) in the presence or absence of conotoxins (100 ng/mL) for osteoclastogenesis. Seven days later, cells were fixed and stained for TRAP activity. Multinucleated TRAP^+^ cells (three or more nuclei) were counted. (B) Human PBMCs were isolated from whole blood and grown with M-CSF (25 ng/mL), hRANKL (25 ng/mL), and conotoxins (100 ng/mL). After 14 d, cells were fixed, stained for TRAP activity, and osteoclasts were quantified. The statistical analysis used one-way ANOVA with Dunnett’s post-hoc test. Scale bar = 500 μm. (C) Mouse bone marrow cells were cultured in the presence of M-CSF (25 ng/mL) and in the presence or absence of RANKL (25 ng/mL) and conotoxins conotoxin (100 ng/mL). After a 7-d differentiation period, cells were fixed and stained for TRAP, followed by observation under a microscope. Scale bar = 500 μm. (D) Mouse bone marrow cells were plated on an Osteo assay plate under osteoclastogenic conditions (M-CSF 25 ng/mL, RANKL 25 ng/mL) in the presence or absence of conotoxins (100 ng/mL). After 6 d of culture, TRAP staining was performed to confirm the presence of osteoclasts. Osteoclasts were removed from the osteo assay plate using a 10% bleach solution, and representative images of each well were captured. The pit areas were quantified using ImageJ software. One-way ANOVA with Dunnett’s post-hoc test. Scale bar = 200 μm.

We then used mouse bone marrow cells and human PBMCs to determine the effect of the conotoxins sXm1b and sVc1.1 on osteoclastogenesis. Mouse bone marrow cells were cultured with RANKL, M-CSF, and conotoxins (100 ng/mL) for 7 d. Osteoclast differentiation was evaluated by staining the cells for TRAP activity. We quantified the number of TRAP^+^ cells that had three or more nuclei. Both conotoxins significantly increased the number of osteoclasts (Figure 4A). We further corroborated the effects of conotoxins on osteoclastogenesis using human cells. Human PBMCs were cultured with RANKL and M-CSF in the presence or absence of sXm1b or sVc1.1 (100 ng/mL) for 9 d. TRAP staining confirmed that both conotoxins significantly increased the differentiation of human osteoclasts (Figure 4B). We sought then to determine whether these conotoxins could induce osteoclast formation without exogenous RANKL or at a suboptimal concentration of RANKL. Mouse bone marrow cells were cultured in the presence or absence of RANKL (25 ng/mL) and conotoxins. In the absence of RANKL, sXm1b, and sVc1.1 (100 ng/mL) alone did not allow the differentiation toward osteoclasts (Figure 4C). However, in combination with RANKL at 25 ng/mL, sXm1b and sVc1.1 increased the number of osteoclasts compared to RANKL alone (Figure 4C). These results suggest that the conotoxins sXm1b and sVc1.1 may not directly induce osteoclast differentiation but enhance RANKL-mediated osteoclastogenesis.

After confirming that conotoxins promote osteoclast formation, we studied conotoxins’ potential to modulate osteoclast resorption activity. We induced the differentiation of mouse bone marrow cells into osteoclasts on Osteo Assay Surface plates for 4 d. Subsequently, osteoclasts were cultured with sXm1b or sVc1.1 conotoxins (100 ng/mL) for 2 d. Following TRAP staining, we quantified the number of osteoclasts to confirm the absence of significant differences between the treated and untreated groups. To evaluate and quantify the resorbed areas, we removed the osteoclasts using a sodium hypochlorite solution (Figure 4D). Our results indicated that both conotoxins, sXm1b and sVc1.1, significantly increased osteoclast resorption pit area, and the percentage of resorbed area compared to the control group (Figure 4D). These findings suggest that the conotoxins sXm1b and sVc1.1 have the potential to enhance bone resorption by stimulating osteoclastogenesis and osteoclast activity.

### Conotoxins regulate bone gene expression in osteoblasts, osteoclasts, and osteocytes

**Figure 5:**
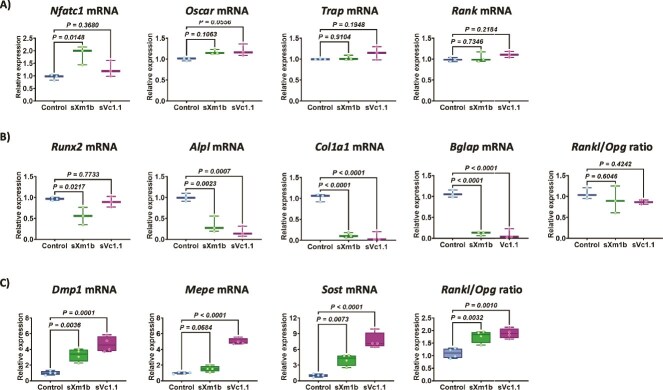
Evaluation of gene expression modulation in bone cells treated with conotoxins by qRT-PCR. Osteoclasts and osteoblasts were differentiated from mouse bone marrow cells and treated with conotoxins. (A) after 7 d of differentiation, we evaluated the expression of *Nfatc1*, *Oscar*, *Trap,* and *Rank* genes in osteoclast cultures. (B) The expression of osteoblast gene markers *Runx2*, *Alpl*, *Col1a1*, *Bglap*, and the *Rankl/Opg* ratio were measured after 21 d of culture. (C) Osteocyte-enriched bone fragments were cultured ex vivo and exposed to conotoxins for 72 h. Total RNA was extracted from each sample, and the expression of marker genes *Dmp1*, *Mepe*, *Sost*, and the *Rankl/Opg* ratio were assessed. Gene expression for all samples was normalized to the housekeeping gene *Rpl32*, and statistical analysis was performed using the Mann–Whitney U test.

Transcriptomic analysis offers valuable insights into the general patterns of gene regulation influenced by conotoxins. Expanding on this knowledge and observing the capacity of conotoxins to regulate the differentiation of osteoblasts and osteoclasts in a controlled environment, we examined the precise modulation of genes involved in bone remodeling using qRT-PCR. In an osteoclastogenesis assay, only sXmb1 increased the expression *Nfatc1* (Figure 5A). Treatment with sXm1b or sVc1.1 did not affect the expression *Oscar*, *Trap*, and *Rank*, which was consistent with the transcriptomic analysis (Figure 5A). In osteoblasts, both sXm1b and sVc1.1 conotoxins resulted in the downregulation of key osteoblast-related genes, *Alpl*, *Bglap*, and *Col1a1* (Figure 5B). The expression of *Runx2* was only decreased by sVc1.1, not by Xmb1 (Figure 5B). There were no statistically significant changes in the *Rankl*/*Opg* ratio in osteoblasts after treatment with the conotoxins sXm1b or sVc1.1 (Figure 5B).

The transcriptomic analysis of osteoblasts treated with conotoxins revealed a decrease in the expression of genes that are likewise expressed by osteocytes. Therefore, to investigate the expression of these genes, we employed osteocyte-enriched bone fragments, cultured them ex vivo, and treated them with conotoxins (100 ng/mL) for 72 h. Our findings indicated that both conotoxins significantly upregulated the expression of *Dmp1*, *Mepe*, *Rankl*, and *Sost* (Figure 5C), critical genes involved in bone remodeling. Significantly, both conotoxins downregulated *Opg* expression, as indicated by the *Rankl*/*Opg* ratio (Figure 5C). These results emphasize the role of sXm1b and sVc1.1 in modulating genes related to bone cells.

### Conotoxins modulate bone remodeling processes in an *ex vivo* bone organ culture

**Figure 6:**
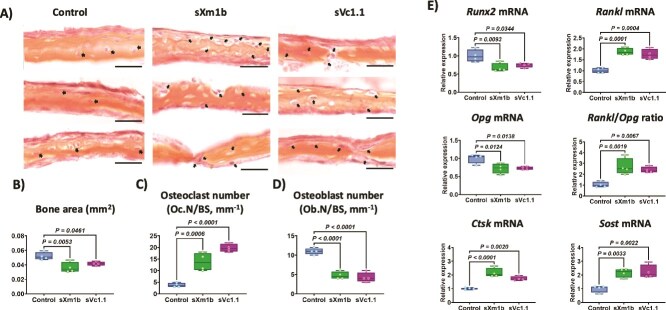
Effect of conotoxins on ex vivo calvaria culture. Hemi-calvarias from newborn mice were cultivated *ex vivo* with or without conotoxins (100 ng/mL) during 7 d. (A) Representative sections of calvariae after H&E staining. Arrows indicate osteoclasts. Scale bar = 100 μm. Histomorphometrical analysis of the bone area (B), normalized number of osteoclasts (C), and osteoblasts (D) on the calvaria. (E) Total RNA from calvaria tissue was used for qRT-PCR to determine the effect of sXm1b and sVc1.1 on the expression of *Runx2*, *Rankl*, *Opg*, *Ctsk*, and *Sost*. *Rpl32* was used to normalize gene expression. Statisticians used the Mann–Whitney U test.

To investigate the effects of the conotoxins sXm1b and sVc1.1 on bone tissue, we used a 3D *ex vivo* calvaria culture model to reproduce the physical and spatial complexity of the bone microenvironment. Ex vivo mouse hemicalvaria were cultivated in the presence of 100 ng/mL of conotoxins over 7 d (Figure 6A). We then assessed changes in bone morphology and gene expression. sXm1b treatment resulted in a reduction in the bone area and an increase in the number of osteoclasts per bone surface (Figure 6B and D), coinciding with the upregulation of *Rankl* and *Ctsk* mRNA expression, which are key contributors to osteoclastogenesis (Figure 6E). The expression of *Opg* and *Runx2* (Figure 6E), crucial regulators of osteoblast differentiation and bone formation, was significantly downregulated. Similarly, conotoxin sVc1.1 induced a decrease in bone surface area and an increase in the number of osteoclasts per bone surface (Figure 6B and C).

Furthermore, sVc1.1 significantly upregulated the mRNA expression of *Rankl* and *Ctsk* while concomitantly downregulating *Opg* and *Runx2* expression (Figure 6E). Additionally, both sXm1b and sVc1.1 promoted the expression of the *Sost* gene (Figure 6E), an important regulator of bone formation. These results collectively suggest that conotoxins sXm1b and sVc1.1 can modulate different aspects of the bone remodeling process.

## Discussion

The bone remodeling process is complex and involves frequent replacement and repair of the bone tissue to maintain skeletal integrity. Deregulation of these processes can lead to a range of debilitating conditions, such as osteoporosis, osteopetrosis, and ectopic bone formation, significantly affecting the quality of life of the patients. Despite available treatments, such as bisphosphonates, hormone therapy, and surgical interventions, limitations in efficacy and long-term safety persist. Therefore, the search for bone remodeling modulators continues.

Conus snail venom contains small peptides called conotoxins. These snails immobilize and trap prey with conotoxins. Due to their affinity and specificity for membrane receptors, transporters, and ion channels, conotoxins are promising drug development candidates. In particular, α-conotoxins are recognized for their affinity for nAChRs, subtype identification, and binding to human receptors.^33^^,^^34^ Current research efforts have been directed toward understanding the impact of nAChRs on bone tissue, with particular emphasis on the α7, α9, and α10 nAChR subtypes. These studies, particularly those employing knockout models, revealed alterations in bone strength and microstructure,^15^^,^^35–37^ suggesting nAChRs may benefit bone health. However, the therapeutic potential of α-conotoxins for bone remodeling is still being explored. This work aims to determine the effects of two synthetic α-conotoxins, sXm1b (α7 nAChR antagonist) and sVc1.1 (α9α10 nAChR antagonist), on bone remodeling.

We first tested conotoxins’ cytotoxic effects on bone marrow cells to verify that therapy did not harm bone cells. Further, we confirmed that α7 and α9α10 nAChRs, sXm1b, and sVc1.1 targets, were expressed in osteoblast and osteoclast cultures in vitro.

Transcriptomic analysis showed that only 19 osteoclast genes were differentially expressed by the conotoxin sXm1b. Compared to sXm1b, sVc1.1 therapy significantly modulated gene expression in osteoclasts, with 443 genes showing significant alterations. GO analysis linked these genes to many biological processes, including cell cycle regulation, proliferation, angiogenesis, vasculature development and cell proliferation. Interestingly, while conotoxins sXm1b and sVc1.1 promoted osteoblast proliferation, our data also indicate a decrease in the expression of genes critical for osteoblast development and differentiation. These findings suggest that conotoxins enhance proliferation by activating the cell cycle and mitosis (G2/M and S phases). They may simultaneously inhibit differentiation, potentially affecting osteoblast functionality. Although we did not observe an apparent modification in the biological processes associated with osteoclast differentiation or activity, these results suggest the potential of conotoxins to modulate gene expression in osteoclast cultures. Other potential mechanisms could explain the differences in the gene modulation by the conotoxins, including biological insensitivity, transcriptional regulation, and protein post-translational modifications, that influence existing proteins’ functions without altering gene expression. Non-transcriptomic factors such as metabolic changes or alterations in cell morphology.

The functional study showed that sXm1b and sVc1.1 increased osteoclast differentiation and resorption in both mouse and human osteoclast models. Further characterization indicated that the conotoxins do not induce osteoclastogenesis independently but instead promote differentiation when combined with RANKL. This suggests that sXm1b and sVc1.1 may interact or enhance RANKL-mediated differentiation pathways, boosting osteoclast differentiation and resorption.

Furthermore, both toxins inhibited osteoblast differentiation and mineralization; however, osteoblast transcriptomics revealed their mechanisms differed. Conotoxin sXm1b downregulated the expression of genes associated with osteoblast differentiation and mineralization, including *Wnt4* and *Wnt9a*, which are associated with osteoblast differentiation through non-canonical pathways,^38^ and collagen genes (*Col15a1*, *Col9a3*, *Col16a1*, and *Pcolce2*). These findings suggest that sXm1b disrupts osteoblast maturation and extracellular matrix formation. The transcriptomic results for osteoblasts treated with sVc1.1 conotoxin showed downregulation of the biological processes related to osteoblast differentiation, mineralization, and skeletal development. In particular, Wnt pathway-related genes (*Wnt4*, *Wnt9a*, and *Wnt10b*, *Fzd9*), bone morphogenic proteins (*Bmp4* and *Bmp7*), and collagen genes (*Col1a1*, *Col1a2*, and *Col11a1*).^39^^,^^40^ This suppression of osteoblast-specific genes suggests an inhibitory effect of sVc1.1 on osteoblast maturation and function, potentially disrupting bone formation.

*In vitro* osteoblast mineralization was significantly reduced by sXm1b and sVc1.1, supporting the transcriptome findings. Quantitative analysis of Alizarin Red staining demonstrated diminished nodule formation and calcium deposition in osteoblast cultures exposed to α-conotoxins, indicating impaired matrix mineralization. qRT-PCR indicated downregulation of osteoblast gene markers, supporting sXm1b and sVc1.1’s inhibitory effects on osteoblast development and function. Interestingly, conotoxin treatment of osteocyte-enriched bone fragments cultivated ex vivo significantly increased the expression of osteocyte differentiation markers *Dmp1*, *Mepe*, *Rankl*, and *Sost*. These genes regulate phosphate homeostasis, mineralization, and maturation, and modulating them may increase osteocyte mineralization to compensate for osteoblast dysfunction.^41^^,^^42^ The findings indicate that conotoxins in the bone may exert a dual influence on the proliferation and differentiation of cellular processes. A more precise mechanism will be clarified by additional research utilizing either normal or mutant osteocyte cell lines to determine the function of a-conotoxin in osteocytes.

In addition, conotoxins can reduce osteoblast differentiation by increasing *Sost* gene expression.^43^ Only the *Rankl*/*Opg* ratio was evaluated in these tissues, not osteoblast gene markers. Osteocytes produce most of RANKL and make osteoclast differentiation and resorption possible. Thus, a higher *Rankl*/*Opg* ratio may indicate a pro-osteoclastogenic bone microenvironment.

Bone remodeling effects of conotoxins were studied ex vivo on mouse hemicalvaria culture models. SXm1b and sVc1.1 treatment increased bone surface osteoclast number and *Ctsk* and *Rankl* gene expression, indicating bone resorption. The transcriptome analysis showed that both α-conotoxins reduced genes involved in osteoblast formation and mineralization. These data support hemicalvaria tissue effects of decreased total bone area, negative *Runx2* expression, and increased *Sost* gene expression. Conotoxins may reduce bone production by downregulating osteoblast function genes, mineralization in vitro, and reducing total bone areas ex vivo. Further studies are needed to determine the precise mechanisms by which the conotoxins influence bone remodeling processes.

In summary, we characterized the effects of 2 synthetic α-conotoxins, sXm1b and sVc1.1, on bone-remodeling processes. Both toxins influenced the activities of osteoclasts and osteoblasts, specifically by promoting osteoclastogenesis and resorption while inhibiting osteoblast development and mineralization. This study establishes a foundation for future investigations into the therapeutic applications of conotoxins in bone disorders, particularly those linked to excess, such as pharmaceutical control of osteoclast activity, coupling abnormalities, and systemic influences like endocrine and metabolic problems. While also advising of possible adverse impacts on bone in patients using conotoxin treatment for pain control.

## Supplementary Material

2025-02-02_Suplementary_Table_S1_ziaf025

## Data Availability

Data supporting this study are openly available from Short Read Archive database (SRA) of the National Center for Biotechnology Information (NCBI) access number PRJNA1108864.
