# Supplementary material for: Conotoxins from sea snails as potential bone remodeling disruptors
Source: JBMR Plus. 2025 Feb 10;9(8):ziaf025. doi: 10.1093/jbmrpl/ziaf025 (PMC12245161; doi:10.1093/jbmrpl/ziaf025)
Supplement: 2025-02-02_Suplementary_Table_S1_ziaf025 [file 2025-02-02_suplementary_table_s1_ziaf025.docx]

**Supplementary information- JBMRPLUS-06-24-0102**

**Table S1. Transcriptomic analysis of osteoblast and osteoclast cultures treated with sXm1b and sVC1.1 conotoxins.**

**Osteoblast cell cycle and proliferation regulated genes under treatment with sXm1b**

| **Cell Cycle** | | |
| --- | --- | --- |
| Symbol | Log2FoldChange | (FDR) |
| Foxm1 | 1.21537443 | 0.0825 |
| Ccnb2 | 1.882033926 | 0.0146 |
| Ccnf | 1.332520325 | 0.0855 |
| Ccn3 | 1.321599165 | 0.036 |
| Cdk15 | 4.860746604 | 0.0055 |
| Cdkl1 | -1.2977334 | 0.021 |
| **Cell proliferation** | | |
| Symbol | Log2FoldChange | (FDR) |
| Mcm5 | 1.459726749 | 0.0725 |
| Aurkb | 1.741773214 | 0.024 |
| Aurka | 1.460516568 | 0.0532 |
| Prc1 | 2.419276264 | 0.0118 |
| ki67 | 2.187067074 | 0.0141 |
| Birc5 | 1.545311357 | 0.061 |
| Kif14 | 2.655730643 | 0.0587 |
| Kif18b | 1.980253875 | 0.0123 |
| Kif15 | 1.950437731 | 0.0424 |
| Kif2c | 1.699989952 | 0.0333 |
| Kif4 | 1.635346626 | 0.0298 |
| Kifc1 | 1.513365873 | 0.0128 |
| Kif11 | 1.407873998 | 0.0888 |
| Cdca3 | 1.45048543 | 0.05 |
| Cdca8 | 1.365683609 | 0.0297 |
| Ska1 | 2.992786681 | 0.0573 |
| Ska3 | 1.892670617 | 0.0122 |
| Wnt4 | -1.8365381 | 0.0615 |
| Wnt9a | -1.3128928 | 0.0533 |
| Serpinf1 | -1.7224802 | 0.0981 |
| Il1b | -1.9939175 | 0.0121 |
| Sox5 | -4.4084153 | 0.0536 |
| Sox6 | -2.343209 | 0.00801 |
| Sox9 | -1.3555309 | 0.00921 |
| Trim72 | -3.0587573 | 0.0374 |
| Trim66 | -2.6690238 | 0.00639 |

**Osteoblast cell cycle and proliferation regulated genes under treatment with sVc1.1**

| **Cell Cycle** | | |
| --- | --- | --- |
| Symbol | Log2FoldChange | (FDR) |
| Cdk15 | 5.55956408 | 0.000319 |
| Cdkn3 | 1.95790172 | 0.0216 |
| Cdkn2c | 1.90835545 | 0.00242 |
| Cdk1 | 1.20991119 | 0.0156 |
| Cdkn1c | 1.20309912 | 0.00000756 |
| Cdk18 | 1.05656461 | 0.00173 |
| Ccnb2 | 2.43668844 | 0.000257 |
| Ccna2 | 2.33498674 | 0.00593 |
| Ccnb1 | 2.14690943 | 0.00819 |
| Ccnf | 2.05399674 | 0.00108 |
| Ccn3 | 1.52126215 | 0.00464 |
| E2f2 | 1.50073155 | 0.0857 |
| E2f7 | 1.45560282 | 0.0905 |
| Aurkb | 2.34782222 | 0.000366 |
| Aurka | 2.07438736 | 0.000937 |
| Cdc20 | 1.63413795 | 0.000092 |
| Cdc45 | 1.55104963 | 0.0212 |
| Cdc6 | 1.46803787 | 0.0724 |
| Cdc7 | 1.39990462 | 0.0418 |
| Cdc25b | 1.31504863 | 0.0121 |
| Foxm1 | 1.77405305 | 0.00191 |
| Chek1 | 2.0879199 | 0.00995 |
| Cdkl1 | -1.6791354 | 0.000561 |
| **Cell proliferation** | | |
| Symbol | Log2FoldChange | (FDR) |
| Mki67 | 2.78398326 | 0.000321 |
| Birc5 | 2.332776114 | 0.000592 |
| Bcl2a1a | 1.5914415 | 0.083 |
| Aunip | 2.33798994 | 0.0479 |
| Kif14 | 3.36463007 | 0.00411 |
| Kif15 | 2.7096535 | 0.000762 |
| Kif18b | 2.59531926 | 0.000143 |
| Kif2c | 2.36320241 | 0.000457 |
| Ccn4;Wisp1 | -2.3536671 | 0.0716 |
| Wnt4 | -2.6326186 | 0.00122 |
| Wnt10b | -2.4496042 | 0.00351 |
| Wnt9a | -1.9492902 | 0.000569 |
| Serpinf1 | -3.4119095 | 0.0000356 |
| Serpine2 | -1.7552819 | 0.00000494 |
| Il1b | -2.9909917 | 0.0000119 |
| Ntrk2 | -4.6432711 | 1.13E-09 |
| Sox6 | -4.2451504 | 5.81E-07 |
| Sox9 | -1.950389 | 0.0000147 |
| Trim72 | -5.0321831 | 0.000406 |
| Trim66 | -2.4507569 | 0.00521 |

**Osteoclast cell cycle and proliferation regulated genes under treatment with sVc1.1**

| **Cell Cycle** | | |
| --- | --- | --- |
| Symbol | Log2FoldChange | (FDR) |
| Foxm1 | 1.54454255 | 0.00636396 |
| Aurkb | 2.56728177 | 0.00283125 |
| Aurka | 2.42988022 | 0.01643823 |
| Ccna2 | 4.20581576 | 0.00053036 |
| Ccnb1 | 3.9519366 | 2.714E-05 |
| Ccnb2 | 3.61679609 | 1.0821E-05 |
| Ccne1 | 1.27846816 | 0.03524139 |
| Cdkn3 | 3.64838198 | 0.0055243 |
| Cdk1 | 2.04003775 | 0.00077263 |
| Cdkn2c | 1.43388685 | 0.01169275 |
| Cdc20 | 2.46856506 | 9.4693E-05 |
| Cdc25b | 1.21456952 | 0.00047705 |
| E2f | 2.89124138 | 0.02548443 |
| E2f2 | 1.03798038 | 0.00345794 |
| Rad51ap1 | 5.25300909 | 0.00206973 |
| Rad54l | 4.79601625 | 0.0023717 |
| Nr4a1 | -2.9197169 | 0.0724 |
| Inhba | -2.1119962 | 0.023 |
| Ccn2 | -1.972561 | 0.0045 |
| Ccn1 | -1.8915784 | 0.00353 |
| Timp3 | -1.7108051 | 0.013 |
| **Cell proliferation** | | |
| Symbol | Log2FoldChange | (FDR) |
| ki67 | 2.945317412 | 0.001696398 |
| Dmkn | 1.24554499 | 0.000192922 |
| Cdca8 | 2.145156876 | 0.000364511 |
| Kif20a | 11.30599399 | 0.027934175 |
| Kif20b | 5.172526451 | 0.005349301 |
| Kif2c | 4.33134579 | 5.60059E-06 |
| Kifc1 | 4.30694789 | 0.018648133 |
| Kif18b | 4.106344071 | 0.000122091 |
| Kif14 | 3.784865902 | 0.006022024 |
| Kif15 | 3.708318764 | 0.000970661 |
| Kif4 | 3.528536386 | 6.64256E-05 |
| Kif11 | 3.395825424 | 0.000128675 |
| Kifc5b | 2.952086818 | 0.056601177 |
| Kif22 | 2.940492852 | 0.00037743 |
| Kif18a | 2.559676624 | 0.005038475 |
| Kif23 | 2.523790056 | 0.00037743 |
| Tnk2 | 4.511735687 | 0.044223066 |
| Osmr | -1.838235385 | 0.00932 |
| Osm | -1.403498545 | 0.00219 |
| Foxp1 | -1.194341389 | 0.00146 |
| Serpine1 | -1.7564434 | 0.00346 |
| Serping1 | -1.7361669 | 0.0539 |
